# Robotic Assistance Enables Inexperienced Surgeons to Perform Unicompartmental Knee Arthroplasties on Dry Bone Models with Accuracy Superior to Conventional Methods

**DOI:** 10.1155/2013/481039

**Published:** 2013-06-19

**Authors:** Monil Karia, Milad Masjedi, Barry Andrews, Zahra Jaffry, Justin Cobb

**Affiliations:** MSK Lab, Department of Orthopaedics, Charing Cross Hospital, Imperial College London, Fulham Place Road, London W6 8RF, UK

## Abstract

Robotic systems have been shown to improve unicompartmental knee arthroplasty (UKA) component placement accuracy compared to conventional methods when used by experienced surgeons. We aimed to determine whether inexperienced UKA surgeons can position components accurately using robotic assistance when compared to conventional methods and to demonstrate the effect repetition has on accuracy. Sixteen surgeons were randomised to an active constraint robot or conventional group performing three UKAs over three weeks. Implanted component positions and orientations were compared to planned component positions in six degrees of freedom for both femoral and tibial components. Mean procedure time decreased for both robot (37.5 mins to 25.7 mins) (*P* = 0.002) and conventional (33.8 mins to 21.0 mins) (*P* = 0.002) groups by attempt three indicating the presence of a learning curve; however, neither group demonstrated changes in accuracy. Mean compound rotational and translational errors were lower in the robot group compared to the conventional group for both components at all attempts for which rotational error differences were significant at every attempt. The conventional group's positioning remained inaccurate even with repeated attempts although procedure time improved. In comparison, by limiting inaccuracies inherent in conventional equipment, robotic assistance enabled surgeons to achieve precision and accuracy when positioning UKA components irrespective of their experience.

## 1. Introduction

Although the benefits of robotic systems in terms of alignment and positioning compared to conventional methods are well established in experienced users [1], the effect of surgical experience and training on the ability to accurately position components with robotic systems is unknown. Conventional unicompartmental knee arthroplasties (UKAs) exhibit a learning curve whereby repetition and experience can lead to improvements in surgical technique, timing, and accuracy [2, 3]. Rees et al. in 2004 demonstrated that a surgeon's UKA performance is significantly worse in their first 10 cases compared to their subsequent 10 cases [3]. Other studies have shown a nonsignificant improvement in accuracy with experience indicating that conventional UKAs have a long learning curve and that even with experience and training obtaining accurate results is difficult [2]. In contrast early results of a preliminary study by Coon demonstrated that the MAKO robotic system may demonstrate a shorter learning curve and greater accuracy compared to conventional techniques [4]. By comparing their first 36 robot assisted UKA patients to their previous 45 conventional UKA patients, they showed that robotic surgery resulted in a posterior tibial slope accuracy that was 2.5 times better and a varus alignment that was 3.2° better than the conventional group. 

Although there are reports of long-term survivorship following UKAs [5] as well as good kinematics [6] and function [7], others have reported a high early failure rate [8]. A variety of factors including patient selection [9] and implant design [10, 11] have been identified as predictors for revision or reoperation of the implant. Incorrect alignments of the tibial and femoral components when performing a UKA have led to poor functional results, high implant wear, and a high revision rate [10–13]. The UKA procedure therefore appears to be more technically demanding, so despite being theoretically both cheaper and better than total knee replacement, its adoption may be limited by surgical skills. Robotic technology has facilitated more accurate and bone preserving methods of UKAs [1, 12, 13] compared to conventional methods which produce inconsistent alignment results [10, 14]. In 2006, Cobb et al. compared the accuracy of Acrobot—a surgical robotic system—to conventional methods of performing a UKA. They showed that all of the 13 robot treated patients had a tibiofemoral coronal alignment within ±2° of the plan, whereas only 9 out of 15 patients treated by conventional means had achieved this accuracy (*P* = 0.001) [1]. By providing computer assistance, the spatial locations of the tools and the patient can be tracked and depicted against a preoperatively created plan on a computer screen which is used by the surgeon as guidance. The plan consists of a three-dimensional (3D) computer model of the patient's bone upon which the ideal position of the prosthesis can be determined and placed on the software. It defines regions within which the robot is constrained to avoid cutting critical areas and to facilitate accurate component placement [1]. This mechanism may enable surgeons to perform accurate UKAs in the early stages of their learning curve when inaccurate placement using conventional methods is most likely [3].

The aims of this novel research were twofold:

1. To assess the accuracy with which surgeons inexperienced in UKAs implant the components using robotic assistance compared to conventional instrumentation.
2. To assess the effect repetition has on component positioning accuracy in both groups.

We surmised that with robotic assistance surgeons inexperienced in UKA will position components consistently and accurately at every attempt, while with conventional instruments component positioning will be inaccurate and improve with more attempts. 

## 2. Methods

### 2.1. Subjects

Sixteen surgeons consented to take part in the study, none of whom had experience in UKAs by neither conventional nor robotic means. Subjects underwent randomisation to one of two groups: conventional UKA or robotic UKA. Each subject performed a UKA once per week for three consecutive weeks by their allocated method on dry bone models. The models used were computer tomography (CT) based replicas of a patient's arthritic knee consisting of a capsule, replica ligaments, and muscle (Medical Models Ltd., London). 

Prior to randomization, a CT scan of the dry bone model used in the study was taken and was segmented using the previously validated Stanmore Implants Modeller Software (Stanmore Implants Worldwide (SIW), Elstree, UK) [15]. A plan of the ideal implant positions on the dry bones was created using the Stanmore Implants Planner (SIW, Elstree, UK) which recreated the joint line and was measured to size 3 and size 4 femoral and tibial Corin Uniglide implants, respectively. The plan was created by a consultant surgeon experienced in UKA and computer-assisted orthopaedic technologies and was designed to be anatomically optimal and achievable using the conventional cutting jig according to the published operative technique. 

 Subjects in the conventional group were instructed to recreate the plan using the Corin Uniglide UKA standard cutting jigs and instruments. A training video was made to show the group how to perform the procedure correctly prior to their first attempt. Additionally, a conventional UKA operating technique instructional booklet was produced based on the Corin Uniglide operative technique and the preoperative plan, which subjects read prior to the procedure and also referred to during the procedure. The guide detailed the steps the subjects needed to follow in order to achieve component placement that recreated the plan. Subjects in the robotic group were shown a demonstration of the UKA procedure using the Sculptor RGA (Stanmore Implants Worldwide, Elstree, UK) (formerly Acrobot) and were also presented with a robotic UKA guide detailing the methodology.

### 2.2. Data Collection and Analysis

Subjects in both groups were timed during the procedure starting with the initial tibial incision to the insertion of the mobile bearing device. All subjects were provided with feedback in between each repeat detailing the accuracy with which they had implanted the components in their previous attempt.

Once the UKA was complete, the bones were separated into the tibial and femoral parts with the Corin Uniglide implants attached and scanned using a 3D laser scanner which provided a computer generated image of the implanted bone. The completed UKAs were coregistered to their respective plan using the 3-matic software (Materialise, Belgium) [16]. This was initially done visually and then fine-tuned using the 3-matic surface matching function by a researcher blinded as to which group each bone model belonged to. The position of the components on the tibia and femur was then compared to that of the ideal plan by recording the coordinates of four points on the planned implant versus the achieved implant. Using Matlab software, local frames of reference of the planned implants were created and compared to those of the achieved implants in all six degrees of freedom (DoF). 

The NextEngine 3D scanner (CA, USA) was used to scan the bones. It is reported to have an accuracy of 0.127 mm and a maximum of 15 samples (points) per millimetre [17]. To validate the accuracy of our methodology, a repeatability study was carried out. Intraobserver reliability involved five repeat measurements of the same bone from which the standard deviation (SD) of the mean translational and rotational errors was reported. Interobserver reliability involved two measurements of the error in six DoF of four randomly chosen bones by two observers from which a Bland-Altman plot was made. The average root mean squared (RMS) differences of three points between the CT-based and laser scanned original bones were also assessed.

The compound rotational errors (calculated as the square root of the sum of the magnitude of the axial, flexion-extension, and coronal alignment errors) and compound translational errors (calculated as the square root of the sum of the magnitude of the medial-lateral, anterior-posterior, and superior-inferior errors) were calculated for each subject for both tibial and femoral components at attempts one, two, and three. A student's *t*-test was used to compare the difference in mean compound rotational and mean compound translational errors between groups at each attempt for each component. A repeated measures ANOVA was used to determine if there was any change in component error within each group between attempts one, two, and three. A post-hoc Bonferroni correction was used for any significant results. Analysis of procedure time was performed by the same statistical methods.

 For each subject their RMS error in each of the six DoF was averaged over their three attempts. This was used to calculate the mean RMS error in each DoF for the robot and conventional group using the data from all three attempts combined. We could then compare this mean absolute error from the plan in each of these DoFs between the robot and conventional groups using a student's *t*-test.

 All statistics were analysed with Statistical Package for Social Sciences 20 (SPSS 20, Chicago, IL, USA), with statistical significance designated as *P* < 0.05.

## 3. Results

**Figure 1:**
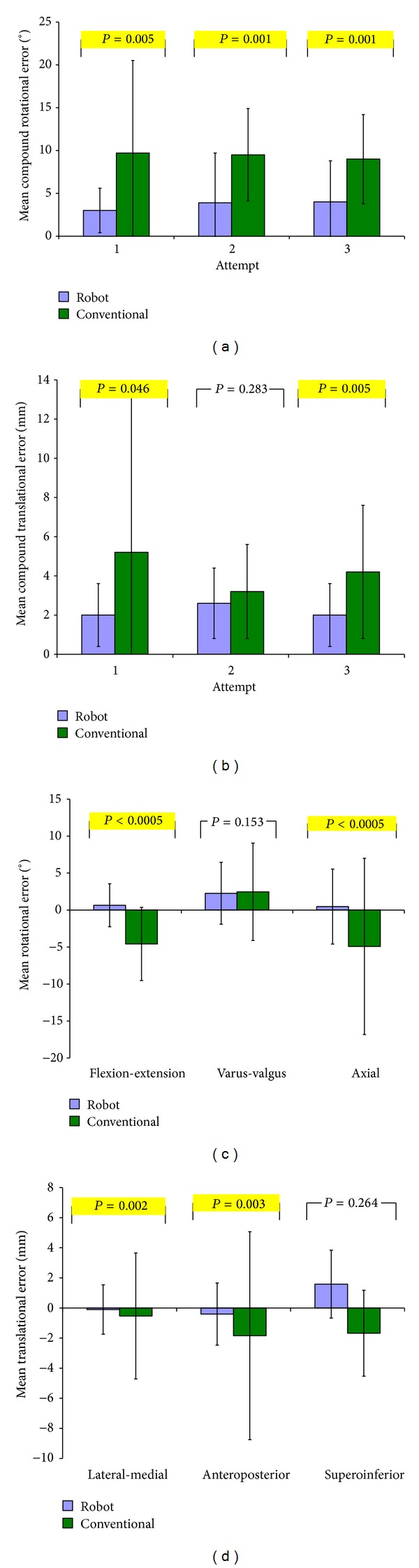
Bar graphs comparing tibial component positioning in robot and conventional groups at attempts 1, 2, and 3 by mean (a) compound rotational error, (b) compound translational error, (c) rotational alignment in each DoF, and (d) translational alignment in each DoF. *P* values compare mean root mean squared errors between groups.

**Figure 2:**
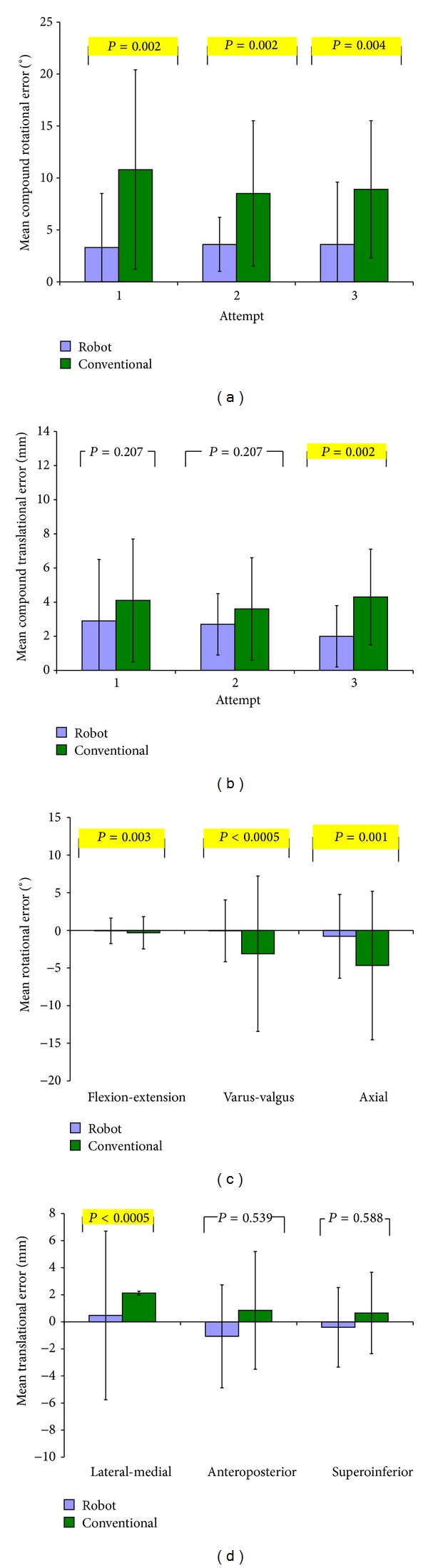
Bar graphs comparing femoral component in robot and conventional groups at attempts 1, 2, and 3 by mean (a) compound rotational error, (b) compound translational error, (c) rotational alignment in each DoF, and (d) translational alignment in each DoF. *P* values compare mean root mean squared errors between groups.

Mean compound rotational error of the tibial component was lower in the robot group compared to the conventional group at all attempts. This difference reached significance at attempts one (3.0° versus 9.7°) (*P* = 0.005), two (3.9° versus 9.5°) (*P* = 0.001), and three (4.0° versus 9.0°) (*P* = 0.001) (Figure 1(a)). The compound translational error was also lower with robotic assistance, reaching significance at attempts one (2.0 mm versus 5.2 mm) (*P* = 0.046) and three (2.0 mm versus 4.2 mm) (*P* = 0.005) (Figure 1(b)). Mean compound rotational error of the femoral component was lower in the robot group, reaching significance at attempts one (3.3° versus 10.8°) (*P* = 0.002), two (3.6° versus 8.5°) (*P* = 0.002), and three (3.6° versus 8.9°) (*P* = 0.004) (Figure 2(a)) as was compound translational error, although this reached significance at attempt three only (2.0 mm versus 4.3 mm) (*P* = 0.002) (Figure 2(b)). 

For the tibial component the robotic group had a lower absolute error in each of the three rotational (axial, sagittal, and coronal) and translational (medial-lateral, anterior-posterior, and superior-inferior) DoF compared to the conventional group. This difference failed to reach significance in only the varus-valgus and superoinferior directions. The mean RMS tibial rotational error was 1.8° ± 1.6° for the robot group compared to 4.7° ± 3.2° (*P* = 0.0002) for the conventional group, while the mean RMS tibial translation error was 1.0 mm ± 0.7 mm for the robot group and 2.1 mm ± 1.5 mm (*P* = 0.021) for the conventional group.

For the femoral component the robotic group had a lower absolute error in each of the three rotational (axial, sagittal, and coronal) and translational (medial-lateral, anterior-posterior, and superior-inferior) DoF compared to the conventional group. This was significant in all DoF except for the superoinferior and anteroposterior directions. The mean RMS femoral rotational error was 1.7° ± 1.7° in the robot group compared to 4.7° ± 3.4° (*P* < 0.0005) in the conventional group, while the mean RMS femoral translation error was 1.3 mm ± 1.0 mm for the robot group and 2.0 mm ± 1.3 mm (*P* = 0.042) for the conventional group.

**Figure 3:**
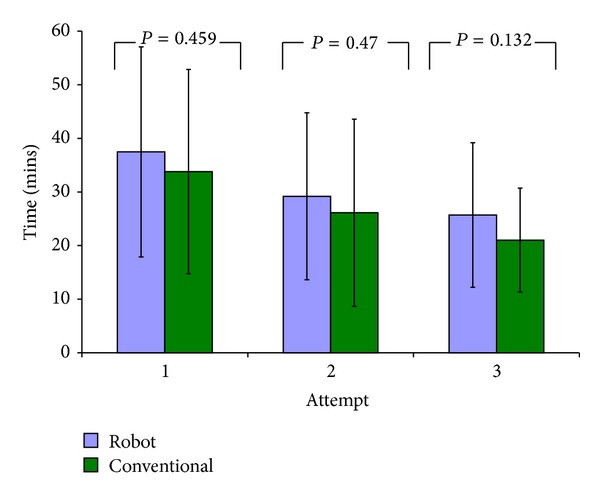
Bar graph showing the mean UKA procedure time at each attempt for the robot and conventional groups. *P* values refer to intergroup analysis. Significant *P* values are highlighted. Error bars = ± 2SD.

Mean procedure time decreased significantly for both robot (37.5 mins to 25.7 mins) (*P* = 0.002) and conventional (33.8 mins to 21.0 mins) (*P* = 0.002) groups with repeated attempts (Figure 3); however, neither group showed a corresponding significant change in rotational (conventional *P* = 0.943, Sculptor RGA *P* = 0.724) or translational (conventional *P* = 0.373, Sculptor RGA *P* = 0.184) component accuracy between attempts.

The results of the intraobserver repeatability study found the mean rotational error of the five repeat bones to be 0.45° ± 0.40° and mean translational error to be 0.23 mm ± 0.15 mm.

**Figure 4:**
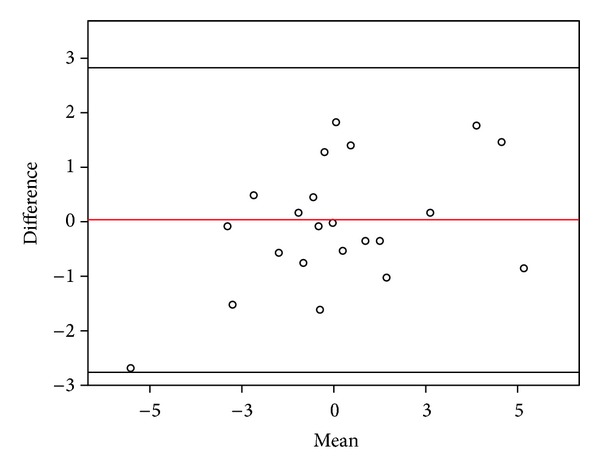
Bland-Altman plot of two observer's agreement of component alignment for five different bones. Red line = mean difference, Black lines = ± 1.96 SD.

The results of the interobserver repeatability study found the mean difference in observation between the two observers to be 0.07 ± 1.39 for each DoF. All measured differences were within ±1.96 SD of the mean difference and hence were within the acceptable limits of agreement (Figure 4). 

RMS errors between the three points on the planned CT bone and laser scanned bone were less than 1 mm for both the femur and tibia.

## 4. Discussion

This randomised study is the first to compare the ability of surgeons to perform accurate UKAs in their initial attempts using both robotic and conventional methods. We have shown that surgeons inexperienced in UKA are able to position components on dry bones when performing a UKA procedure significantly more accurately with the Sculptor RGA than by conventional methods alone and can do so repeatedly and without any prior experience. We have also used a novel method in assessing the accuracy of component positioning in dry bone models which seem robust when judged by our repeatability studies.

The goal of any instrumentation used in arthroplasty should be to allow its user to position the components in a position and orientation which is preoperatively or intraoperatively determined. While there is no precise agreement on the ideal position of implants during a UKA, correct alignment of the femoral and tibial components has been shown to be the most objectively quantifiable factor in determining the wear and longevity of UKAs [12, 18]. This is particularly relevant in the early stages of a surgeon's learning curve when improper component placement is more likely due to the difficulty of the procedure and the relatively little exposure surgeons have to UKAs [3].

In our study, the decrease in time exhibited by both groups between attempts signifies the presence of a learning curve; however, the conventional group did not demonstrate a corresponding increase in accuracy in either rotational or translational alignment between attempts, while robot assistance ensured that accurate placement was consistently produced at each and every attempt. The lack of improvement in accuracy in the conventional group highlights the need for timely feedback for surgeons in training if they are to produce consistently accurate results. Although we were unable to show any increase in accuracy with repetition in this study, the variability of the component positioning was the highest in the conventional group's first attempt for both tibial (Figure 1) and femoral (Figure 2) components compared to following attempts. This suggests that the precision of component positioning may improve with time, although the study was neither designed nor powered to detect this. 

Accuracy of the compound rotational alignment of the tibial component was consistently more accurate than conventional methods over all three attempts. Compound rotational alignment consists of axial rotation, coronal rotation, and the posterior slope, of which the latter is the most reported alignment measure dictating outcomes of a UKA procedure, and as a result posterior slopes greater than 7° should be avoided [12]. The 7° slope built in the conventional jig did not prevent any of the subjects from producing a tibial component placement posterior slope of >7° with errors ranging from +0.5° to +12.6°. Other robotic systems with experienced users have also demonstrated improved sagittal tibial component placement, including the Acrobot [1] and the MAKO robot [4]. This concurs with our results, which showed a significant difference in the magnitude of posterior slope error between the robot group (1.2° ± 1.0°) and conventional groups (4.6° ± 2.5°) (*P* < 0.0005). 

Although compound translational errors were higher in the conventional group at every attempt, this was only significant at attempts one and three for the tibial component (Figure 1(b)) and attempt three for the femoral component (Figure 2(b)). Considering the individual femoral translational DoFs only the medial-lateral translation showed a significant difference, while superoinferior and anteroposterior errors were similar between the two groups (Figure 2(d)). This agrees with previous findings which also found similar results between the robot and conventional groups with experienced users in these DoF [1]. This may be due to the instrumentation used in a conventional UKA. The tibial stylus improves depth control when resecting the tibial plateau which dictates the inferosuperior component error explaining the similar mean robot (2.0 mm ± 0.9°) and conventional (1.7° ± 0.9°) errors. During femoral preparation the small reamer can be set to an accuracy of 1 mm, the result of which dictates the superoinferior positioning of the component. Comparatively, the rotational alignment and medial-lateral translational alignment of both components as well as anteroposterior alignment of the tibial component rely on referencing of bone landmarks, and as a result the instrumentation may lack precision. This can be compared to the TKA procedure whereby the location of transepicondylar axis has been shown to have an interobserver discrepancy of 23° [19], while intraobserver error and interobserver error of 6° and 9°, respectively, in the identification of the epicondylar axis have also been shown [20, 21]. Analogously these discrepancies would exist in a UKA procedure when attempting to align the femoral and tibial jigs using guidelines relying on the patient's anatomy. This reflects our results and others [1] and explains significant differences found in most, but not all, directions of alignment in the conventional group compared to the robot group.

Overall our results of errors in the positioning and orientation of both components using the Sculptor RGA were comparable to results of experienced surgeons operating on real patients: Dunbar et al. [22] found that the MAKO robot's mean RMS errors for the tibia were 1.4 mm and 2.6° and for the femur 1.2 mm and 2.1°, while Cobb et al. [1] reported mean RMS errors using the Acrobot to be 1.1 mm and 2.5° for the tibia and 1.0 mm and 2.6° for the femur. Our robot results using inexperienced UKA surgeons on dry bones are comparable: mean RMS errors of 1.0 mm and 1.8° for the tibial component and 1.3 mm and 1.7° for the femoral component indicate that novice robot users can reproduce experienced surgeons' results. Our slightly lower values may be due to errors introduced during cementing in vivo, which has been reported to give errors of up to 2° in UKAs [23]. 

We recognise several inherent limitations of our study. It is a small study, using only 3 repetitions to demonstrate learning so may miss an improved performance later in the learning curve, although the largest improvement might be expected to be early in the experience. We did not demonstrate this. It is also a dry bone study. However, the dry bones were replicas of a patient's arthritic tibia and femur with replica ligaments and a capsule attached and hence were as realistic to a real patient as possible. The fact that our accuracy results were comparable to published in vivo data supports the validity of the dry bone model. However, the use of dry bones prevents reproduction of soft tissue balancing and the selection of an appropriate thickness of bearing. Therefore, measurement of the tibiofemoral angle is meaningless. Although this is an important measure of functional outcome following a UKA, component alignment is a major influence of tibiofemoral angle [24, 25], thus justifying the conclusions.

 Arthroplasty requires precision and accuracy to be delivered consistently for favourable outcomes. Robotic systems have repeatedly demonstrated superiority over conventional methods when used by experienced users. We have demonstrated that this level of exactitude can be replicated on a dry bone model by surgeons who are unfamiliar with the procedure. Robotic technology, in the form of the Sculptor RGA, enables surgeons to perform this demanding form of arthroplasty accurately without prior experience. It achieves this by removing the inaccuracies inherent in the use of conventional instrumentation.
